# Evaluation of a bioengineered ACL matrix’s osteointegration with BMP-2 supplementation

**DOI:** 10.1371/journal.pone.0227181

**Published:** 2020-01-07

**Authors:** Paulos Y. Mengsteab, Patrick Conroy, Mary Badon, Takayoshi Otsuka, Ho-Man Kan, Anthony T. Vella, Lakshmi S. Nair, Cato T. Laurencin

**Affiliations:** 1 Connecticut Convergence Institute for Translation in Regenerative Engineering, University of Connecticut Health, Farmington, CT, United States of America; 2 Raymond and Beverly Sackler Center for Biological, Physical and Engineering Sciences, University of Connecticut Health, Farmington, CT, United States of America; 3 Department of Orthopedic Surgery, University of Connecticut Health, Farmington, CT, United States of America; 4 Department of Biomedical Engineering, University of Connecticut, Storrs, CT, United States of America; 5 Department of Immunology, University of Connecticut School of Medicine, Farmington, CT, United States of America; 6 Department of Materials Science and Engineering, University of Connecticut, Storrs, CT, United States of America; 7 Department of Chemical and Biomolecular Engineering, University of Connecticut, Storrs, CT, United States of America; 8 Department of Reconstructive Sciences, University of Connecticut Health Center, Farmington, CT, United States of America; Università degli Studi della Campania, ITALY

## Abstract

A poly (l-lactic) acid bioengineered anterior cruciate ligament (ACL) matrix has previously demonstrated the ability to support tissue regeneration in a rabbit ACL reconstruction model. The matrix was designed for optimal bone and ligament regeneration by developing a matrix with differential pore sizes in its bone and ligament compartments. Building upon past success, we designed a new bioengineered ACL matrix that is easier to install and can be used with endobutton fixation during ACL reconstruction. To achieve this, a new braiding procedure was developed to allow the matrix to be folded in half, making two-limbs, while maintaining its bone and ligament compartments. The osteointegration of the matrix with and without bone morphogenetic protein 2 (BMP-2) supplementation was evaluated in a rabbit ACL reconstruction model. Two doses of BMP-2 were evaluated, 1 and 10 μg, and delivered by saline injection into the bone tunnel at the end of surgery. A fibrous matrix-to-bone interface with occasional Sharpey’s fibers was the primary mode of osteointegration observed. The matrix was also found to support a fibrocartilage matrix-to-bone interface. In some cases, the presence of chondrocyte-like cells was observed at the aperture of the bone tunnel and the center of the matrix within the bone tunnel. Treatment with BMP-2 was associated with a trend towards smaller bone tunnel cross-sectional areas, and 1 μg of BMP-2 was found to significantly enhance osteoid seam width in comparison with no BMP-2 or 10 μg of BMP-2 treatment. Regenerated tissue was well organized within the bioengineered ACL matrix and aligned with the poly (l-lactic) acid fibers. Disorganized tissue was found between the two-limbs of the bioengineered ACL matrix and hypothesized to be due to a lack of structural scaffolding. This study suggests that the bioengineered ACL matrix can undergo similar modes of osteointegration as current autografts and allografts, and that BMP-2 treatment may enhance osteoblastic activity within the bone tunnels.

## Introduction

Annually, 400,000 anterior cruciate ligament (ACL) reconstructions are performed worldwide to repair ruptured ACLs. Overall, the outcomes of ACL reconstruction are satisfactory with 90% of patients achieving normal or nearly normal knee function [1]. However, 30% of young patients reinjure their knee following ACL reconstruction [2], and only 63% return to their preinjury activity level [1]. The gold standard grafts for ACL reconstruction are hamstring tendon and bone-patellar tendon-bone autografts and allografts [3]; autografts have the drawback of donor site morbidity and allografts have the potential for disease transmission and graft rejection [4]. To overcome these drawbacks, research has focused on developing bioengineered matrices for ACL regeneration [5].

We have previously demonstrated that a bioengineered ACL matrix could support the regeneration of the ACL in a rabbit model [6]. The bioengineered ACL matrix was a braided construct made of multifilament yarns of poly (l-lactic) acid (PLLA). The braided matrix design consisted of an interconnected porous structure with appropriate pore sizes to support bone and soft tissue regeneration. Furthermore, the braided matrix was designed to have similar mechanical properties to the native rabbit ACL matrix. In a rabbit ACL reconstruction model, the bioengineered ACL matrix demonstrated dense collagen tissue ingrowth and vascularization at 12-weeks. This seminal study focused on the intra-articular regeneration of the scaffold but did not investigate the osteointegration of the bioengineered ACL matrix.

Osteointegration of a bioengineered ACL matrix, or any ACL graft, is critical, as the lack of osteointegration may lead to bone tunnel widening and later anterior instability of the knee [7, 8]. One of the first methods employed to enhance osteointegration was to wrap an autograft or allograft with a collagen sponge that was soaked in bone morphogenetic protein-2 (BMP-2), a clinically relevant growth factor used for bone repair [9, 10]. BMP-2 is an osteoinductive growth factor that has been shown to significantly enhance bone formation *in vitro* and *in vivo* [11–16]. BMP-2 signaling enhances the expression of *runx2*, *dlx5*, and *osterix* which promote the condensation of mesenchymal stem cells, proliferation of osteoprogenitor cells, and differentiation of immature osteoblasts to mineralized osteoblasts and finally to osteocytes [11–16]. Rodeo et al. demonstrated that wrapping an autograft with a collagen sponge soaked in BMP-2 enhanced pull-out loads from the bone tunnel in an extraarticular bone tunnel healing model [9]. Furthermore, it was demonstrated that the fibrous tissue interface was reduced, suggesting greater osteointegration between the tendon and bone.

The aim of this study was to evaluate the osteointegration of a bioengineered ACL matrix with and without the supplementation of BMP-2. Several methods of BMP-2 delivery were considered including the use of a gel carrier, wrapping the matrix with a collagen sponge soaked in BMP-2, or a saline injection. Injection with a gel carrier was deemed to be difficult to control and wrapping with a collagen sponge would add excess bulk to the matrix. Therefore, we hypothesized that an injection of BMP-2 in saline would enhance the osteointegration of the bioengineered ACL matrix. BMP-2 was delivered to the femoral and tibial tunnel at the end of an ACL reconstruction via a saline injection. The bioengineered ACL matrix was designed with two-limbs (double graft) to facilitate the use of suspension fixation using an endobutton on both the tibial and femoral sides, which has demonstrated stronger graft fixation than compression fixation [17]. The regeneration of tissue with and without BMP-2 was assessed at 12-weeks, and two doses of BMP-2 were evaluated, 1 and 10 μg. Finally, the long term osteointegration of the bioengineered ACL matrix without BMP-2 supplementation was evaluated at 24-weeks.

## Material and methods

### Bioengineered ACL matrix fabrication

Poly(l-lactic) acid (PLLA) yarns were obtained from Teleflex Medical OEM (Coventry, CT). Twelve PLLA 60 denier multifilament fibers were twisted together to form PLLA yarn (720 denier). To produce the bioengineered ACL matrix, twenty-four yarns were braided into a 4 x 4 square braid design using a custom-built row and column braiding machine [18]. The 4 x 4 design is characterized by 4 rows and columns of bobbins in the center, 16 bobbins in total. Additionally, 8 bobbins surrounded the center 4 x 4 matrix to facilitate the braiding motion. Two braiding heights were used to braid the bioengineered ACL matrix, 37 cm and 67 cm, measured from the tip of the bobbin carriers to the braiding point above the carrier. A rotating collector was used to collect the braided structure and was manually controlled to ensure that the braiding point was consistent throughout the process. The lower braiding point height of 37 cm was used to develop the boney region of the bone, and a braiding point height of 67 cm was used for the intra-articular portion of the braid. Throughout the braiding process, the braiding height was manually adjusted to achieve the boney and intra-articular region morphology needed for the bioengineered ACL matrix design.

### Mechanical testing

An Instron P5200 with a 2 kN load cell and pneumatic action grips for cord and yarns (Instron 2714–040) was used for tensile tests. The bioengineered ACL matrix length inserted in the machine was 37 cm, the clamp pressure was set to 40 psi, and a 2% strain rate (mm/s) was applied based on the measured gauge length. The single graft (n = 4) and double graft (n = 3) were both tested. Data was processed from the load vs. extension curve to determine the load at failure, yield load, stiffness, Young’s Modulus, and yield stress. The unpaired t-test was used for statistical analysis. Raw mechanical data can be found in S1 File.

### Bioengineered ACL matrix preparation and surgical procedure

Animal experiments were approved by the Institutional Animal Care and Use Committee at the University of Connecticut Health. Thirty-six New Zealand white rabbits (12-weeks old, 3–4 kg) were divided into three groups (n = 9, each) for a 12-week time point: (1) bioengineered ACL matrix, (2) +1 μg BMP-2, and (3) +10 μg BMP-2. Nine additional animals received a bioengineered ACL matrix without BMP-2 and were euthanized at 24-weeks. Pre-operative analgesic was administered (Buprenorphine). Anesthesia was administered with a cocktail of ketamine, xylazine, and atropine. Isoflurane was utilized for anesthesia maintenance.

**Fig 1:**
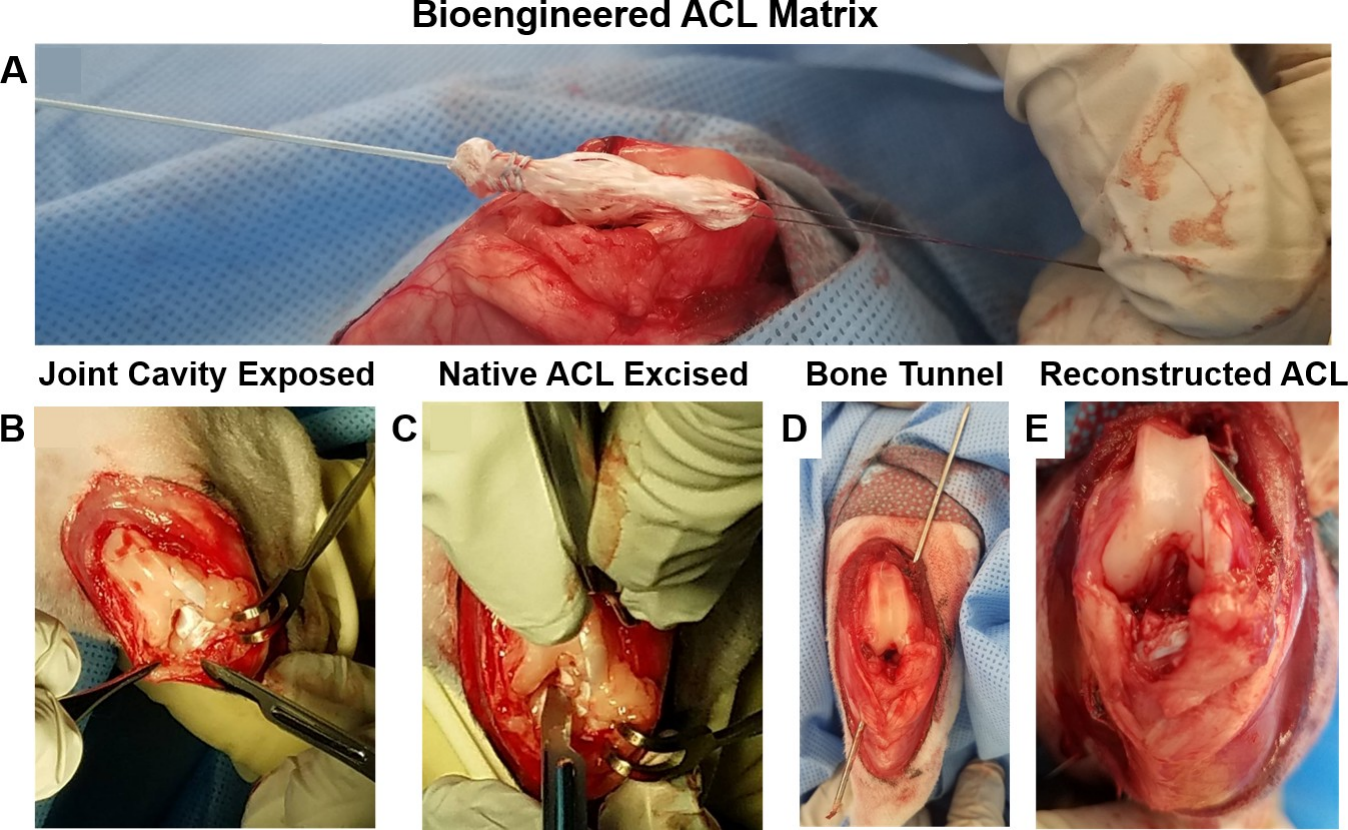
Surgical schematic of the ACL reconstruction procedure. A) Demonstration of the bioengineered ACL matrix prior to insertion into the femoral and tibial bone tunnels. B) Para-patellar arthrotomy approach demonstrating the exposure of the joint cavity. C) Demonstration of the excision of the native ACL. D) Demonstration of the transtibial bone tunnel. E) Visualization of the reconstructed ACL, which is anchored by titanium suture buttons.

Prior to surgery, bioengineered ACL matrices were plasma treated for 1 minute at 0.5 Torr and 100 Watts. The matrices were then double bagged in autoclave pouches, sterilized with ethylene oxide, and stored in a desiccator. During surgery, the two limbs of the bioengineered ACL matrix were sutured together with 2–0 fiberloop (Arthrex) with three throws. Additionally, 2–0 fiberloop was passed through the looped end of the bioengineered ACL matrix and threaded through the eyelet of a needle (Fig 1A).

The operation was performed on the left knee for all rabbits. A vertical midline longitudinal incision was made extending from the distal femur to the tibia. The skin and subcutaneous fascia were retracted to expose the patellar tendon. The dissection was continued between the quadriceps tendon and the vastus medialis muscle. The capsule and the synovial membrane were divided from the inner border of the patella and the patellar tendon. With the knee in extension, the patella was dislocated, but not inverted. Subsequently, the knee was flexed to expose the joint cavity. To expose the ACL, the fat pad was dissected with a midline incision but left intact (Fig 1B). The ACL was excised at its tibial attachment, and the stump was removed (Fig 1C). Using a 1.1 mm k-wire, the femoral bone tunnel was created through the anatomic footprint of the ACL and exited the lateral femoral cortex (Fig 1D). The angle of the bone tunnel was approximately 45 degrees from the long axis of the femur. Subsequently, a collinear bone tunnel was created in the tibia using the same k-wire with the knee in 30 degrees of flexion. A 3.0 mm cannulated drill bit was placed over the k-wire and used to dilate the bone tunnels. Finally, the bone tunnels were flushed with saline.

The bioengineered ACL matrix was then passed through the tibia and then the femur. The looped end was positioned in the femur and secured to a 3.5 mm titanium suture button (Arthrex). With the knee in full extension and the matrix in manual maximal tension, the suture from the whipstitch was secured to a titanium suture button on the tibial cortex with a knot (Fig 1E). A movie of the surgical procedure is shown in S1–S7 Movies. The knee was subsequently tested for stability and proper implantation with the Lachman test. Post-operatively, the rabbits received daily administration of antibiotics (Baytril) for three days. Post-operative analgesics were given in the form of fentanyl patches and were removed after three days.

### Blood collection and cytokines quantification

Pre-operatively, blood was collected from each rabbit. Blood was also collected on day 2, 7, 14, 28, 42, 56, and 84 days for rabbits in the 12-week time point groups (n = 6). For the 24-week time point, blood was also collected at 112 and 168 days (n = 6). The rabbits were sedated with acepromazine prior to blood collection. Blood was collected from either the marginal ear vein or the central artery and placed in K2-EDTA collection tubes (BD Vacutainer). Plasma was collected using Ficoll-Paque density gradient media (GE Healthcare) following the manufacturer’s procedure. If the volume of blood was limited, the ratio of Ficoll medium to blood was maintained. An equal volume of Hank’s balanced salt solution was added to the blood and mixed. Subsequently, Ficoll-Paque medium was added to a 15 mL falcon tube and blood was carefully layered on top at a ratio of (3:4). Samples were then centrifuged at 400 g for 40 minutes at 18°C. The upper layer containing plasma and platelets were transferred to Eppendorf tubes and frozen at −80°C. The concentration of the cytokines was measured using a Quantibody Rabbit Cytokine Array following manufacturer’s instructions (Catalog#: QAL-CYT-1, RayBiotech, Norcross, GA). One-way ANOVA with Dunnett post-hoc analysis was conducted at each time point to determine significance between treatment groups. One-way ANOVA with Dunnett post-hoc analysis was conducted to determine the significance of time within each treatment group. GraphPad Prism 8.2.0 was used for all statistical analysis. Raw cytokine data can be found in S2 File.

### Micro-CT (μCT) analysis

At 12- and 24-weeks rabbits were euthanized by an overdose of ketamine. Knee joints were harvested and fixed in 10% neutral buffered formalin. After 24 hours, the knee joints were placed in fresh formalin. Formalin was changed every 3 days up to 14 days. At day 14, samples were removed from formalin and rinsed with running tap water overnight. Samples were then dehydrated in a series of alcohols and cleared in xylene: 70% ethanol (2 days), 95% ethanol (2 days), 100% 2-propanol (2 days), xylene (2 days). The reagents in the dehydration and clearing step were changed every 24 hours. When the samples were incubated in 70% ethanol, they were imaged and analyzed using the vivaCT 40 μCT system (Scanco Medical, Switzerland). Scan settings were at 55 kV, 90 mA, and spatial resolution at 23 mm. Contours of the bone tunnel shape were made every ten slices; the entirety of the bone tunnel was used for analysis. When bone did not surround the bone tunnel in any given cross-sectional slice it was excluded from the analysis. Forward iterations were used to create contours that matched the shape of the bone tunnel (three iterations per contour, contrast boundary of 55 to 600). The contours were then morphed together, and the volume of the bone tunnel was analyzed through a preset script of the vivaCT 40 software. The average cross-sectional area of the tunnel was determined by the following equation:

$$Average\;bone\;tunnel\;\mathrm{cross}‐\mathrm{sectional}\;area=\frac{Bonet\;tunnel\;volume}{\#of\;slices*slice\;thickness}$$ (1)

One-way ANOVA with Dunnett post-hoc analysis was conducted to determine significance between tibial and femoral bone tunnel cross-sectional areas at 12 weeks. An unpaired t-test was conducted to determine significance between 12- and 24-week samples. GraphPad Prism 8.2.0 was used for all statistical analysis. Raw computed CT data can be found in S3 File.

### Histological staining and analysis

After dehydration, samples were embedded in methyl methacrylate following the procedure outlined by Erben [19]. Goldner’s staining was achieved by following the procedures outlined by Villanueva et al. [20]. Toluidine Blue staining was achieved by overstaining the samples in 2% (w/v) Toluidine Blue O diluted in deionized water and then dehydrating in 100% ethanol until the desired level of metachromasia was achieved. For Von Kossa staining, samples were incubated in 5% silver nitrate solution under incandescent light for 30 min. Subsequently, the samples were treated with 5% sodium thiosulfate for 3 min to remove remaining silver nitrate, then counterstained with azophloxine. All stained sections were cleared in xylene for approximately 10 min prior to mounting. Images of sections were obtained using a Leica DMi8 microscope at a magnification of 80x or 160x. Osteoid width was measured throughout the bone tunnel. Measurement of fibrous tissue interface was limited to the mid-tunnel region. Three independent rabbit knees were used for the assessment. The femur was only analyzed due to inconsistencies in the tunnel position of the tibia. One-way ANOVA with Dunnett post-hoc analysis was conducted to determine significance between treatment groups. Raw measurement data can be found in S4 File.

### Scanning electron microscopy imaging

After fixation of the knee joints, the samples were placed in 70% ethanol. The bone tunnel was carefully cut open along the sagittal axis with the matrix intact. The matrix was divided into three segments: intra-articular region, mid-tunnel, and tunnel exit region, followed by dehydration in increasing concentration of ethanol (80% to 100%). Samples were then dried with a critical point dryer. Samples were mounted onto stubs and the base coated with silver paint to enhance the conductivity of the sample. Samples were imaged on the FEI Nova NanoSEM 450 system.

## Results

### Mechanical testing of the bioengineered ACL matrix

**Fig 2:**
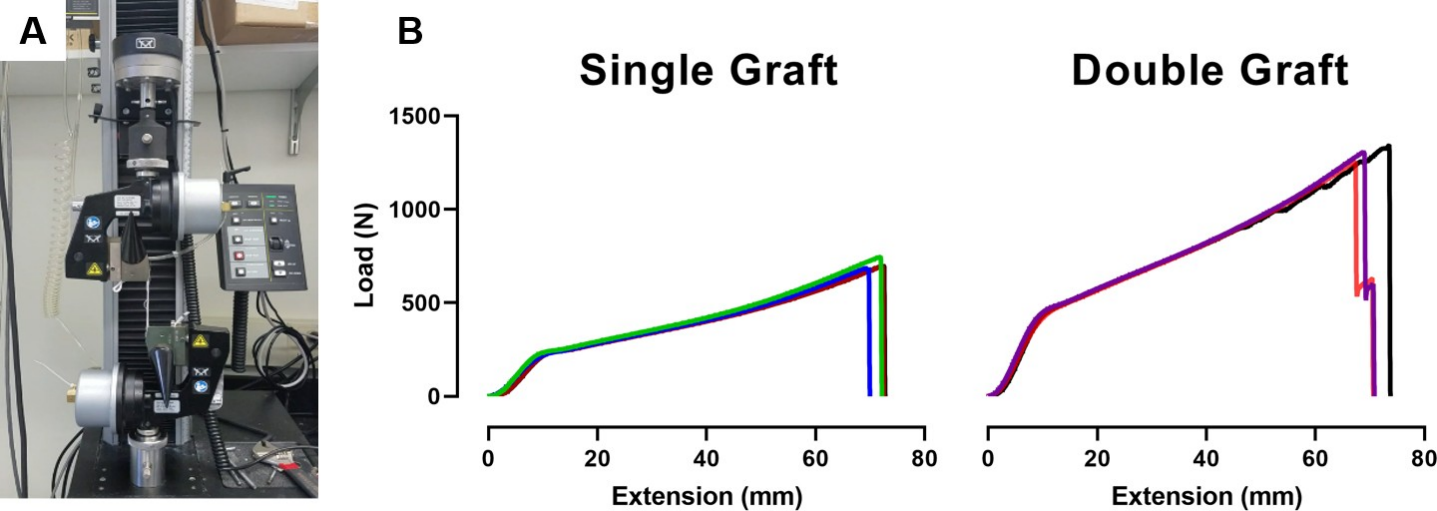
Mechanical testing of bioengineered ACL matrix. A) Demonstration of the mechanical testing setup. The braided structure is folded over, and a pneumatic clamp is used to fix the graft. B) Load vs. extension curve for a single graft and a double graft.

The bioengineered ACL matrix was designed to allow for the ease of insertion and to achieve higher peak loads than the native rabbit ACL, which has been reported to be 314 ± 68 N [6]. To this end, the matrix was designed so that it could be folded in half while maintaining discrete boney and intra-articular regions. Subsequently, the matrix’s tensile strength was tested (Fig 2A). Two bioengineered ACL matrices were fabricated a single graft and double graft. The single graft was 1.4 mm x 1.4 mm (width and thickness). The single graft was folded in half to fabricate a double graft with a dimension of 1.4 mm x 2.8 mm (width and thickness). Peak loads for the single graft and double graft were 707 ± 26 N and 1301 ± 43 N (Table 1, p-value = 0.0001), respectively, and the load versus extension graphs are plotted in Fig 2B. The stiffness of the double graft was twice that of the single graft (62 ± 2.2 N/mm vs. 31 ± 1.9 N/mm, p-value = 0.0001). The load at yield was 218 ± 6.1 N and 430 ± 10.65 N for the single and double graft, respectively (p-value = 0.0001). No statistical difference in the extension at failure, Young’s Modulus, or yield stress was found between the single and double graft as shown in Table 1.

**Table 1:** Mechanical and material properties of the bioengineered ACL matrix.

		Single Graft	Double Graft	P—value
**Peak Load (N)**		707 ± 26	1301 ± 43	0.0001
**Extension at Failure (mm)**	72 ± 1.6	72 ± 1.6	0.7301
**Linear Region Stiffness (N/mm)**	31 ± 1.9	62 ± 2.2	0.0001
**Cross Sectional Area (mm**^**2**^**)**		1.96	3.92	--
**Youngs Modulus (MPa)**		4137 ± 238.4	4022 ± 121.7	0.4826
**Yield Stress (MPa)**		110 ± 3.4	108 ± 4.2	0.6555
**Load at Yield (N)**		218 ± 6.1	430 ± 10.65	0.0001
**Molecular Weight of PLLA (kDa)**	144			
**Crystallinity**	82%			

### Gross surgical outcomes at twelve and twenty-four weeks

**Fig 3:**
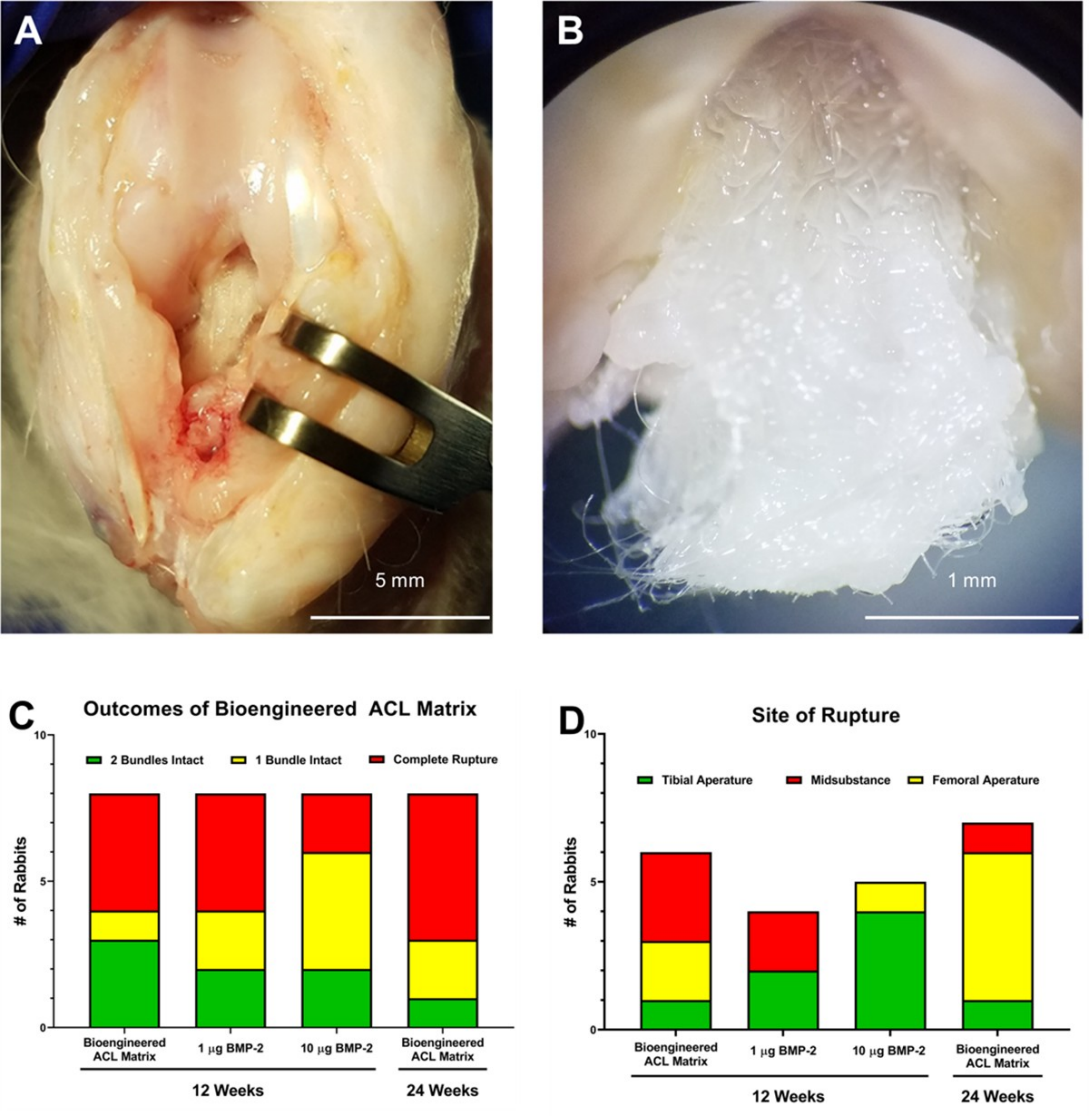
Gross visualization and outcomes of rabbit ACL reconstruction surgery. Gross morphology of intact bioengineered ACL matrix at A) 12-weeks and B) 24-weeks. The femur is on the top, and the lateral condyle is on the right side for A) and B). A) Demonstrates a healthy knee joint with no signs of fibrosis or cartilage erosion. B) is a zoomed in image of the ACL at 24-weeks. The ACL was transected before the image was taken. The glistening nature of the tissue is a sign of healthy tissue. C) Outcomes of the procedure by experimental group demonstrates no significant difference in matrix outcomes between treatment groups. D) Analysis of the site of rupture in the investigated conditions demonstrates a random nature of rupture.

Fig 3A & 3B demonstrate the gross morphology of the bioengineered ACL matrix at 12-week and 24-week time points, respectively. At both time points, the biodegradable fibers of the matrix can be seen. Fibrous tissue growth on the matrix is present at each time point. At the time of harvest, the bioengineered ACL matrix was classified as having both bundles intact, one bundle intact, or a complete rupture (Fig 3C). Supplementation of BMP-2 did not affect if the matrix was intact or not. A Fisher exact t-test did not find any significance between the outcomes of the bioengineered ACL matrix alone in comparison to supplementation with 1 μg or 10 μg of BMP-2. The relative risk for complete rupture in comparison to the bioengineered ACL matrix alone was 1.0 (95% confidence interval: 0.3666 to 2.728) and 0.5 (95% confidence interval: 0.1273 to 1.758), for matrices supplemented with 1 μg or 10 μg of BMP-2, respectively.

Three modes of rupture were observed in the matrices: femoral aperture, tibial aperture, or mid-substance rupture. At 12-weeks, the bioengineered ACL matrix without supplementation of BMP-2 displayed all three types of ruptures (Fig 3D). BMP-2 supplemented groups primarily ruptured at the tibial aperture and mid-substance. At 24-weeks, the bioengineered ACL matrix displayed all types of ruptures.

SEM imaging at 12-weeks was conducted to analyze the mode of material rupture. At 12-weeks, the intra-articular morphology of the ruptured bioengineered ACL matrices demonstrated splitting of PLLA fibers resembling a mop-like appearance (S1 Fig). PLLA fibers also demonstrated blunt ends in some cases. PLLA fibers were ruptured in pure tension *in vitro* and demonstrated a mushroom cap morphology.

### μCT analysis of bone tunnel

**Fig 4:**
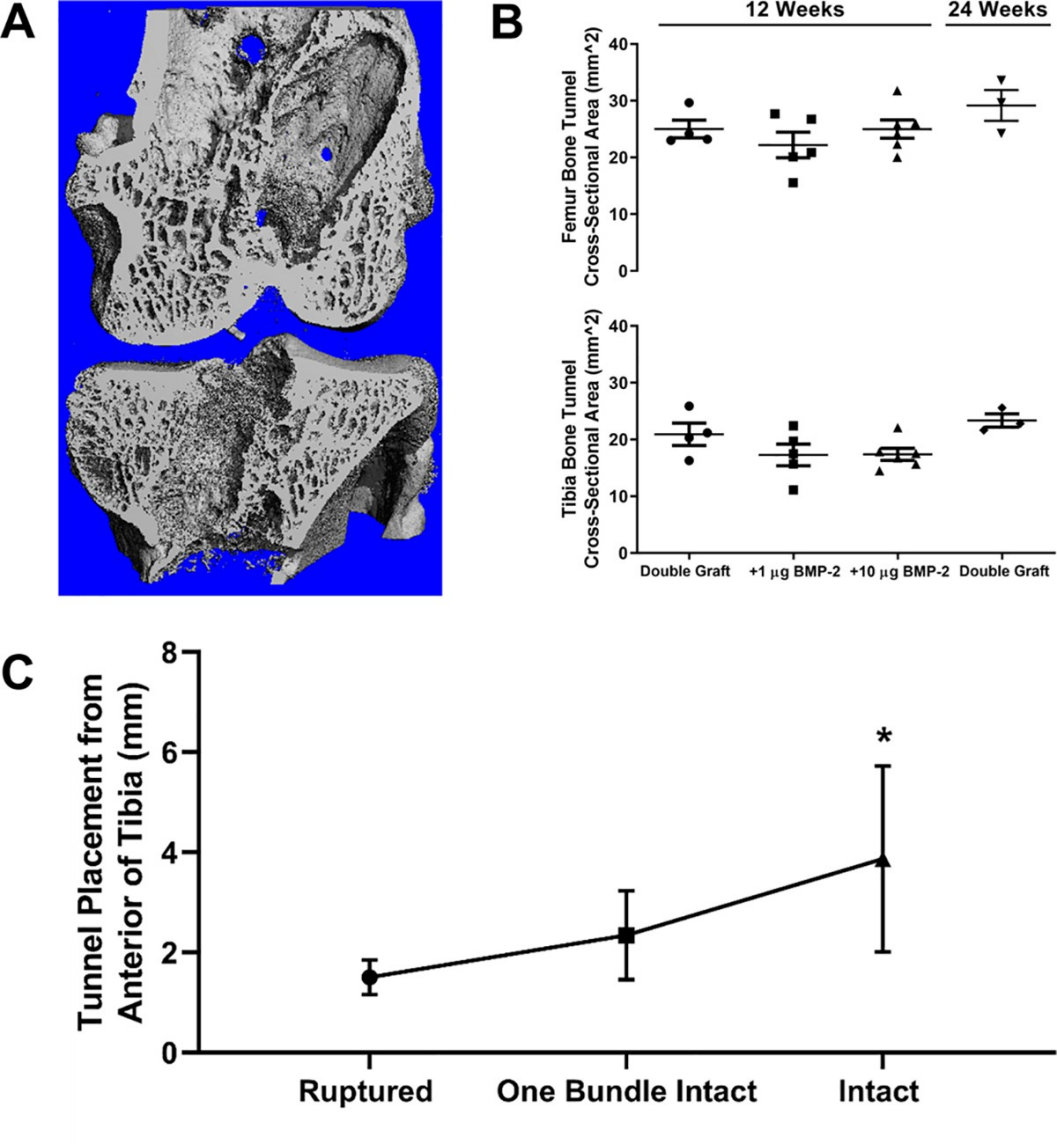
Quantitative analysis of bone tunnel cross-sectional area. A) Representative 3-D coronal section of the femoral and tibial tunnel. B) Quantitation of the cross-sectional area of the femoral and tibial tunnel demonstrates no significant difference between all groups. C) Tunnel placement on the tibia and its relationship to the outcome of the bioengineered ACL matrix.

Micro-CT analysis was conducted to evaluate bone regeneration at 12- and 24-weeks. Bone tunnels did not show signs of bone regeneration in the center of the bioengineered ACL matrix within the bone tunnel (Fig 4A). Supplementations of BMP-2 did not significantly affect the ratio of bone volume to tissue volume or the average tunnel area of the tibia or femur (Fig 4B). Since there was no statistical significance between the groups the data was combined to assess the differences in bone regeneration for the tibia and femur. The average femoral and tibial tunnel cross sectional area was 24.20 ± 4.61 mm^2^ and 19.10 ± 4.05 mm^2^, respectively, and the difference was significant (p-value = 0.0123). Furthermore, the effect of the tibial tunnel position on the outcome of matrix failure was assessed. Distance of the tibial tunnel from the anterior aspect of the tibia to the outer edge of the bone tunnel was measured for ruptured and intact matrices and was found to be 1.5 ± 0.35 mm, 2.3 ± 0.89 mm, and 3.9 ± 1.86 mm for samples that were ruptured, had one bundle intact, and fully intact, respectively (Fig 4C). There was a significant difference in the tibial tunnel position between ruptured and intact matrices (p = 0.004).

### Evaluation of matrix osteointegration

**Fig 5:**
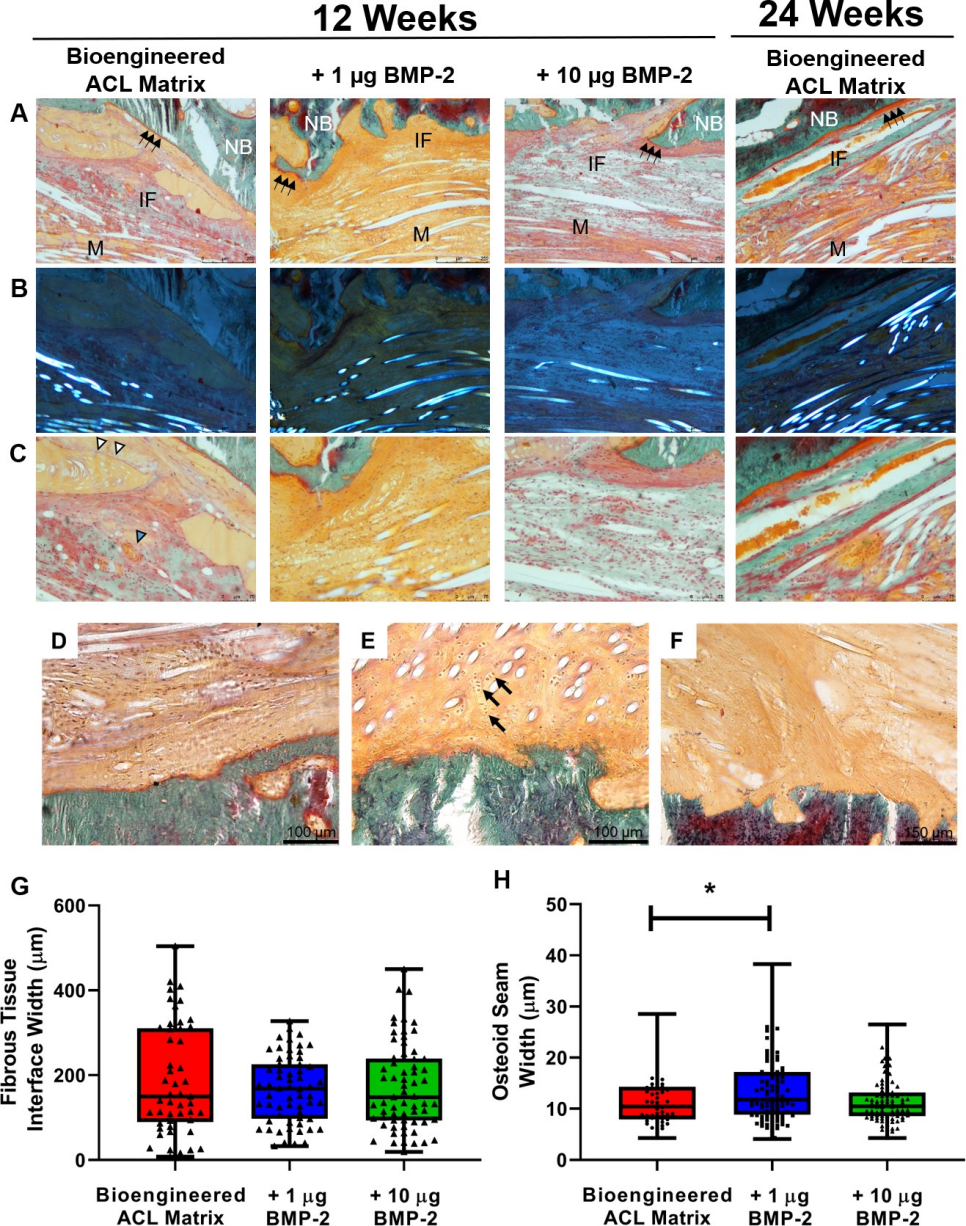
Goldner’s Trichrome stain of the femoral tunnel matrix-to-bone interface and quantification of the interface and osteoid seam width. A) 80x images in the mid-center of the bone tunnel region. The osteoid seam can be seen at the matrix-to-bone interface (green arrows). The turquoise green stain in the matrix-to-bone interface demonstrates newly forming bone. At 24-weeks, greater osteointegration was observed for the bioengineered ACL matrix demonstrated by an increase in mineralized bone at the interface. B) Polarized images of the same region in panel A. The bright fiber or circular structures denote the presence of the PLLA fibers. C) Magnified images of panel A demonstrates the presence of blood vessels (blue arrowhead) and sinusoids (white arrowheads). Different modes of osteointegration seen in bone tunnel (D-F). D) Tissue is found to be parallel to PLLA fibers. E) Fibrocartilage formation with presence of isogenic groups of chondrocytes (black arrows). F) Presence of Sharpey’s fibers. Quantification of the fibrous tissue interface G) and H) osteoid seam width. The mean fibrous tissue interface width for the bioengineered ACL matrix, +1 μg BMP-2 and +10 μg BMP-2 groups are 188.7 ± 129.6 μm, 165.8 ± 78.35 μm, and 172.9 ± 102.2 μm, respectively. The mean osteoid seam width for the bioengineered ACL matrix, +1 μg BMP-2 and +10 μg BMP-2 groups are 11.66 ± 5.203 μm, 13.68 ± 6.24 μm, and 11.51 ± 4.43 μm, respectively. (* = p-value of 0.0406) NB, native bone; IF, interface; M, matrix.

Goldner’s Trichrome staining was carried out to assess the osteointegration of the bioengineered ACL matrix within the bone tunnel. Fig 5A–5C demonstrates the main mode of osteointegration seen in all treatment groups. At 12-weeks, the newly forming osteoid was observed adjacent to the native bone and towards the interface in the mid-center of the bone tunnel (Fig 5A, black arrows). Polarized microscopy of the same field of view demonstrates the PLLA fibers of the bioengineered ACL matrix in respect to the native bone and interface (Fig 5B). At 24-weeks the osteoid band was generally wider. The turquoise green staining in the interface represents mineralized bone formation, which was found consistently in the bioengineered ACL matrix and +10 μg BMP-2 group. Only one sample of three in the +1 μg BMP-2 group demonstrated newly forming mineralized bone in the interface. Von Kossa staining demonstrated light reaction to mineralization in the interface for the +1 and +10 μg BMP-2 group (S2 Fig). Vascularization was present in the interface and includes fenestrated sinusoids and blood vessels (Fig 5C, white and blue arrowheads).

The interface generally showed indirect matrix integration with the bone (Fig 5E). Interfacial tissue organization was anisotropic and in line with the major axis of the bioengineered ACL matrix (Fig 5E). In rare instances, direct matrix integration was seen and defined as isogenic groups of chondrocytes lying in the interface perpendicular to the bioengineered ACL matrix (Fig 5E). In some cases, Sharpey’s fibers could be seen connecting the mineralized bone and matrix (Fig 5F).

Fibrous tissue interface width and osteoid seam width were quantified from Goldner’s Trichrome stained images at 12-weeks. The fibrous tissue interface width of the mid-tunnel region did not demonstrate any significant differences between the bioengineered ACL matrix and the treatment groups (Fig 5G). Bioengineered ACL matrices treated with +1 μg of BMP-2 demonstrated significantly higher osteoid seam width in comparison to the bioengineered ACL matrix (Fig 5H).

### Toluidine blue staining for glycosaminoglycan content

**Fig 6:**
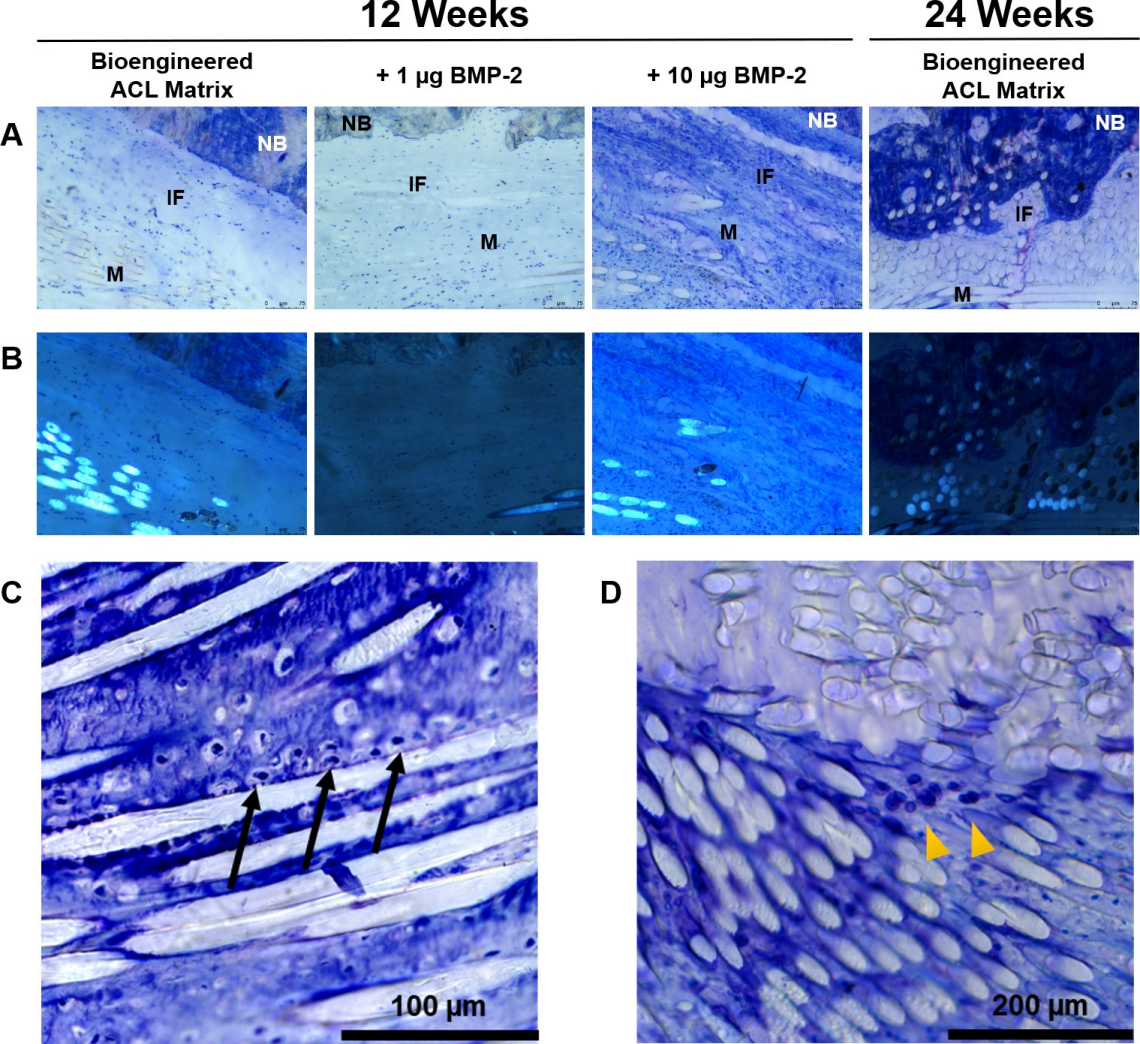
Toluidine Blue stain of the femoral tunnel matrix-to-bone interface. A) 80x images in the mid-center of the bone tunnel region. Minimal proteoglycan was observed at the matrix-to-bone interface in all 12-week samples (proteoglycans stain purple). At 24-weeks, the bioengineered ACL matrix was incorporated with mature bone. B) Polarized images of the same region in panel A. C) Toluidine Blue staining of the aperture of the femoral bone tunnel at 12-weeks. Proteoglycan content was observed (purple staining) and accompanied by the presence of chondrocyte-like cells (black arrows). D) Presence of chondrocyte-like cells (yellow arrowheads) found in the center of the bioengineered ACL matrix. NB, native bone; IF, interface; M, matrix.

Proteoglycan content was assessed throughout the bone tunnel via Toluidine Blue staining. Overall no demonstrative difference was noted between the bioengineered ACL matrix alone and the treatment groups at 12-weeks (Fig 6A). Metachromasia of the Toluidine Blue staining, purple tint, was similar in the interface between groups at 12-weeks. The PLLA fibers of the bioengineered ACL matrix were visualized by polarized light (Fig 6B). At 24-weeks, the integration of PLLA fibers into woven bone was demonstrated by non-polarized and polarized images (Fig 6A & 6B). Furthermore, greater metachromasia was seen in bioengineered ACL matrices implanted for 24-weeks. In most samples, chondrocyte-like cells were seen at the intra-articular-to-bone tunnel interface. The chondrocyte-like cells seem to lie on the PLLA fibers and were surrounded by proteoglycan content, as denoted by the purple staining of Toluidine Blue (Fig 6C). In some cases, chondrocyte-like cells were also found in the bone tunnel proper and away from the matrix-to-bone interface (Fig 6D).

### Tissue organization and cytokine analysis

**Fig 7:**
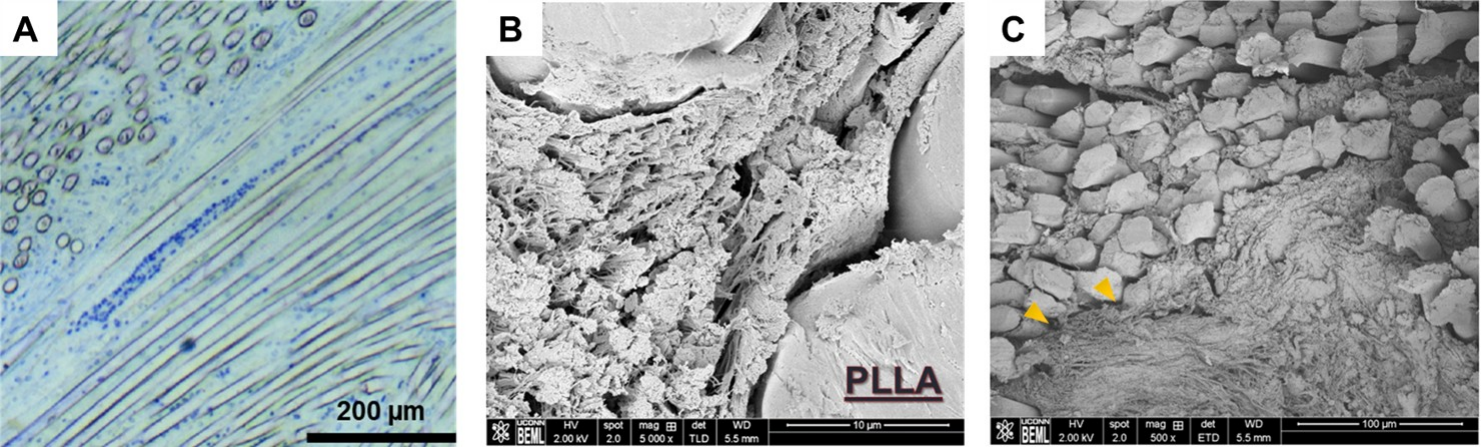
Tissue organization within the bone tunnel. A) Columnar arrangement of fibroblasts. B) SEM image demonstrating anisotropic tissue organization and alignment with PLLA fibers. C) SEM image demonstrating disorganized tissue (yellow arrowheads) between the two limbs of the bioengineered ACL matrix.

The cellular and tissue alignment within the matrix was analyzed to determine the ability of the bioengineered ACL matrix to guide structural organization. Fig 7A demonstrates organization of fibroblast cells that are organized along the major axis of the matrix. Adjacent to the matrix-to-bone interface, nanofibrous extracellular matrix deposition could be seen at 12-weeks (Fig 7B). The nanofibrous extracellular matrix was anisotropic and aligned with the longitudinal axis of the PLLA fibers in the bioengineered ACL matrix. In the center of the matrix, in between the two-limbs, dense nanofibrous extracellular matrix was observed that was isotropic (Fig 7C).

Systemic cytokine levels were measured to assess the healing process. Ten cytokines were measured using a multiplex assay of which only interleukin 8 (IL-8) and macrophage inflammatory protein 1b (MIP-1b) levels were detected within the range of the standard curve (S3 Fig). For the bioengineered ACL matrix, a steady incline of MIP-1b levels were seen from day 42 to 168. This suggests that cytokine levels may be detected in blood plasma that may relate to the progression of ACL healing (S5 File).

## Discussion

Accelerated and enhanced osteointegration after ACL reconstruction allows for early and robust matrix stabilization within the bone tunnels. This may enable earlier rehabilitation of the knee and subsequent return to pre-surgery activity level. Both tendon grafts and bioengineered matrices must achieve osteointegration, or may suffer from bone tunnel enlargement, leading to graft laxity, and eventually anterior instability of the knee. Therefore, we aimed to understand the mechanism of osteointegration for the bioengineered ACL matrix and whether BMP-2 could enhance osteointegration.

In this study, the bioengineered ACL matrix exhibited both direct and indirect osteointegration in the bone tunnel. Direct integration consists of a natural progression from tendon to bone, identical to natural enthesis development [21]. Indirect integration occurs when collagen fibers, called Sharpey’s fibers, project perpendicularly from the bone to anchor the graft [22]. In this study, we saw the formation of Sharpey’s fibers at the matrix-to-bone interface in all groups (Fig 5F). In addition, our histology sections exhibited the presence of sinusoids and small blood vessels at the matrix-to-bone interface indicating the formation of fibrovascular tissue, a precursor to bone ingrowth [23]. This mode of osteointegration has similarities with descriptions of autograft and allograft tendon-to-bone healing [24–27].

To our knowledge, this is the first study to report that a bioengineered ACL matrix can support fibrocartilage growth at the matrix-to-bone interface in an ACL reconstruction model at 12-weeks. This demonstrates that the matrix possesses the ability to support direct integration into bone without the need for cells or growth factors. There are a few reports of fibrocartilage formation at the tendon-to-bone interface after stem cell delivery [27,28], transfection of stem cells with BMP-2 [29], and the use of Mg screws instead of Titanium interference screws [30]. Lim et al. demonstrated that the application of 3 to 4 million mesenchymal stem cells in a fibrin glue gel generated a distinct intervening zone of fibrocartilage between the hamstring tendon and bone in a rabbit ACL reconstruction model [27]. Furthermore, they demonstrated that the failure load of the MSC treated group was significantly higher at 8-weeks. A fibrocartilage tendon-bone junction is desired because it resembles the native integration of the ACL and is thought to contribute to stronger mechanical properties. In our study, the site of fibrocartilage formation was found in the bone tunnel at the matrix-to-bone interface and towards the extra-cortical exit. This positioning suggests that potential cell sources for fibrocartilage formation include mesenchymal progenitor cells from the bone marrow and the periosteum.

Formation of a fibrocartilage zone was not influenced by BMP-2 treatment. However, treatment with 1 μg of BMP-2 enhanced osteoid seam width and demonstrated a trend toward reduced bone tunnel cross sectional area, suggesting enhanced osteoblast activity (Figs 4B and 5H). Nevertheless, the effect of BMP-2 was not robust in respect to boney ingrowth into the bioengineered ACL matrix at 12 weeks. This is likely owed to the lack of a drug carrier used for BMP-2 administration. Hashimoto et al. demonstrated that a saline injection of 15 μg of BMP-2 induced the formation of ossicles in the flexor digitorum communis tendon and the Achilles tendon to promote osteointegration after autograft transfer for ACL reconstruction [31]. Tendons are dense substances and the success of the work done by Hashimoto et al. may be due to the retention of BMP-2 within the tendon. In contrast, the bioengineered ACL matrix we used is porous and likely did not retain the BMP-2. Past studies have used a variety of carriers to localize and sustain the bioactivity of BMP-2 in ACL reconstruction and bone tunnel healing models, including collagen sponges [9], calcium phosphate cement [25,32], and fibrin gels [32,33]. Generally, these studies have indicated that the carrier alone did not enhance bone integration, but the combination with BMP-2 did elicit greater bone integration as defined by enhanced mechanical properties of the interface and the presence of cartilage at the interface. Further investigation utilizing drug carriers with prolonged retention of BMP-2 to enhance osteointegration is needed.

Although a drug carrier may attenuate BMP-2 activity, the lack of biomechanical data in this study limits a definitive conclusion on the ability of BMP-2 saline injections to enhance the fixation of the matrix. Bone tunnel pullout strength is a primary criterion to assess the strength of osteointegration in tendon-to-bone healing models, and the high rate of matrix ruptures in this study prevented biomechanical testing. This led us to investigate the cause of matrix ruptures in this study.

We first investigated the design of the matrix, which was designed for ease of implantation and to facilitate organized tissue infiltration. SEM analysis demonstrated anisotropic tissue deposition within the matrix fibers that were in line with the loading axis, a significant improvement over past ACL prostheses [34]. However, in the center of the matrix, the region between the two limbs, isotropic tissue organization was observed and was likely due to the lack of structural cues. This suggests that a double graft bioengineered ACL matrix design limits anisotropic tissue organization throughout the matrix and may limit its tensile strength. Therefore, future investigations may consider utilizing a single graft design.

Nevertheless, the mechanical properties of the bioengineered ACL matrix exhibited during *in vitro* testing provided confidence in the ability of the matrix to withstand *in vivo* mechanical loads. The peak load of the matrix was four times stronger than that of the native rabbit ACL at the time of implantation. Additionally, the yield load of the matrix, 430 N, was higher than the load at failure of the native rabbit ACL. However, 41% of the matrices were ruptured at 12-weeks, and 63% were ruptured at 24-weeks (Fig 3C). The axial splitting and bushy-end morphology of the ruptured PLLA fibers suggested that that the failure mechanism was flexural and rotational fatigue [35]. However, this early fatigue failure was inconsistent with a previous sheep study utilizing the same biomaterial [36]. Thus, we suspected that the high failure rate was a consequence of the surgical technique used in this study [37–44].

We found that an anterior tibial tunnel position was associated with a higher likelihood of matrix rupture (Fig 4C). We can reason that the anterior tibial tunnel position caused over-tensioning of the matrix throughout knee flexion, which is described by the work of Fleming et al. [39]. Based on our findings, we would recommend positioning the outer edge of the tibial tunnel 4 mm from the anterior aspect of the tibia in future rabbit ACL reconstruction models (Fig 4C). Additionally, rabbit knee physiology needs to be considered when fixing the matrix. At rest, the rabbit knee is at approximately 150° of flexion [38]. In this study, the matrix was fixed at 0° of knee flexion. Thus, the matrix was over tensioned at rest. Future investigations utilizing the bioengineered ACL matrix should control for tunnel position, angle of matrix fixation, and initial tension.

## Conclusion

We investigated the osteointegration of a bioengineered ACL matrix with and without supplementation of BMP-2 delivered through a saline injection. Supplementation of BMP-2 through saline injections into the bone tunnel showed a trend towards reduced cross sectional area. Overall, the effect of BMP-2 was not robust. Different modes of osteointegration were observed with the primary mode being indirect osteointegration with the presence of Sharpey’s fibers. It was found that the bioengineered ACL matrix could support fibrocartilage formation in the bone tunnels as well as the enthesis without BMP-2 treatment. Future studies should investigate the use of different drug carriers for the localized and sustained delivery of BMP-2 to enhance osteointegration of the bioengineered ACL matrix.

## Supporting information

S1 Fig Gross morphology of ruptured samples at 12 weeks in comparison to fibers loaded in axial tension that ruptured *in vitro*. A) Low magnification and B) high magnification view demonstrated brush like morphology as well as blunt ends in the ruptured fibers. The morphology is suggestive of mixed modes of rupture that were reminiscent of polyester fibers that were exposed to biaxial fatigue and buckling failure.
(TIF)

S2 Fig Von Kossa staining of the femoral tunnel matrix-to-bone interface. Black staining represents mineralized bone and the pink-red stain represents connective tissue (azophloxine counterstain). Light mineralization can be seen in the interface of the +1 and +10 μg group, and reduction of the fibrous tissue interface can be seen in the 24-week group.
(TIF)

S3 Fig **Evaluation of systemic IL-8 A) and MIP-1b B) levels before and after ACL reconstruction.** (σ = significant difference for 10 μg group in comparison to pre-operative levels; γ = significant difference between control and 1 μg BMP-2; # = significant difference between control and 10 μg BMP-2; Φ = 1 ug group analysis between pre-operative levels; * = control pre-operative vs time point cytokine values).
(TIF)

S1 Movie Surgical procedure—Part 1. (MP4)

S2 Movie Surgical procedure—Part 2. (MP4)

S3 Movie Surgical procedure—Part 3. (MP4)

S4 Movie Surgical procedure—Part 4. (MP4)

S5 Movie Surgical procedure—Part 5. (MP4)

S6 Movie Surgical procedure—Part 6. (MP4)

S7 Movie Surgical procedure—Part 7. (MP4)

S1 File Raw mechanical data. (XLSX)

S2 File Raw cytokine data. (XLSX)

S3 File Raw computed CT data. (XLSX)

S4 File Raw data for fibrous tissue interface and osteoid seam measurements. (XLSX)

S5 File Discussion on cytokine data. (DOCX)
