# Supplementary figures and images for: Evaluation of a bioengineered ACL matrix’s osteointegration with BMP-2 supplementation
Source: PLoS One. 2020 Jan 7;15(1):e0227181. doi: 10.1371/journal.pone.0227181 (PMC6946545; doi:10.1371/journal.pone.0227181)

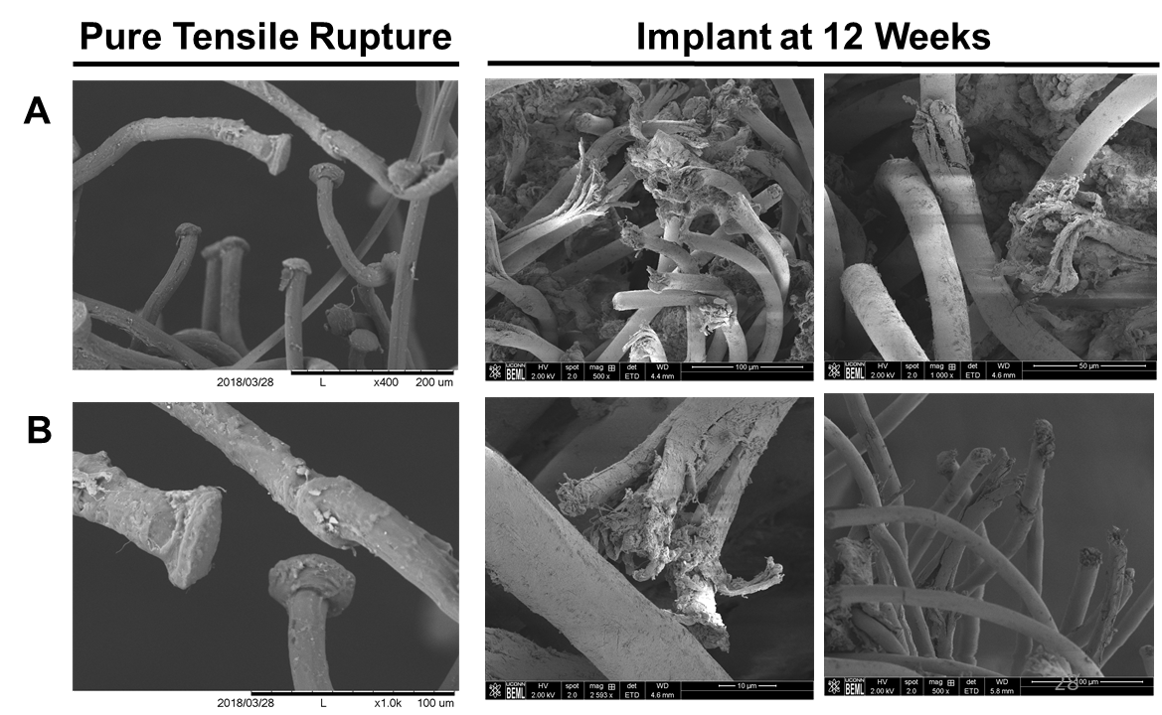

Supplement: S1 Fig — A) Low magnification and B) high magnification view demonstrated brush like morphology as well as blunt ends in the ruptured fibers. The morphology is suggestive of mixed modes of rupture that were reminiscent of polyester fibers that were exposed to biaxial fatigue and buckling failure. (TIF) [file pone.0227181.s001.tif]

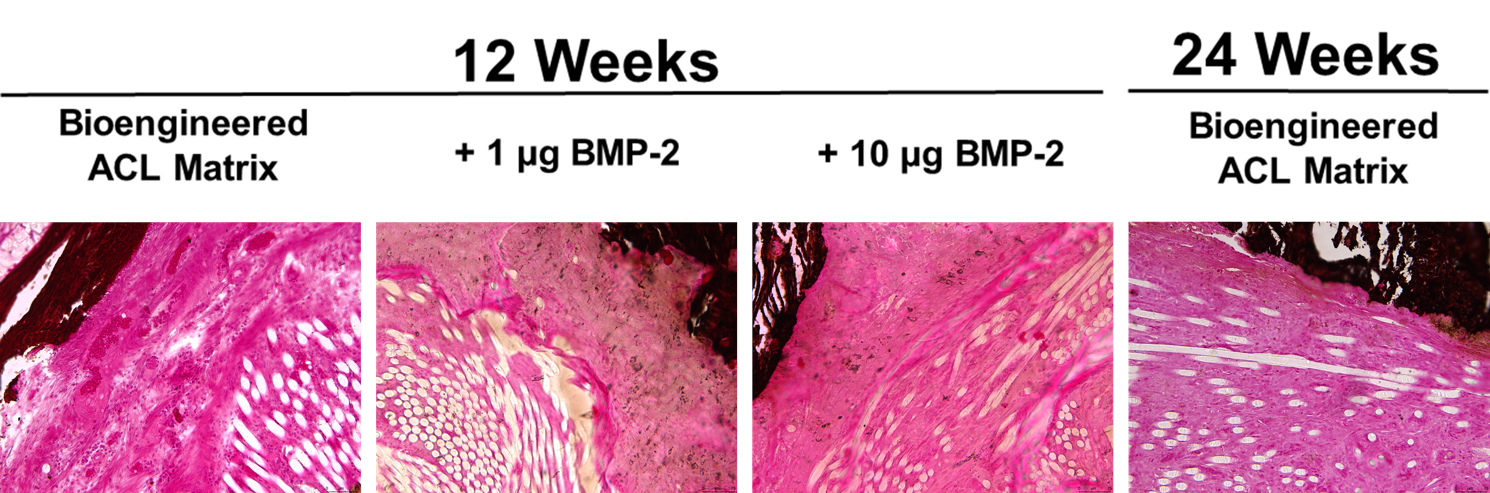

Supplement: S2 Fig — Black staining represents mineralized bone and the pink-red stain represents connective tissue (azophloxine counterstain). Light mineralization can be seen in the interface of the +1 and +10 μg group, and reduction of the fibrous tissue interface can be seen in the 24-week group. (TIF) [file pone.0227181.s002.tif]

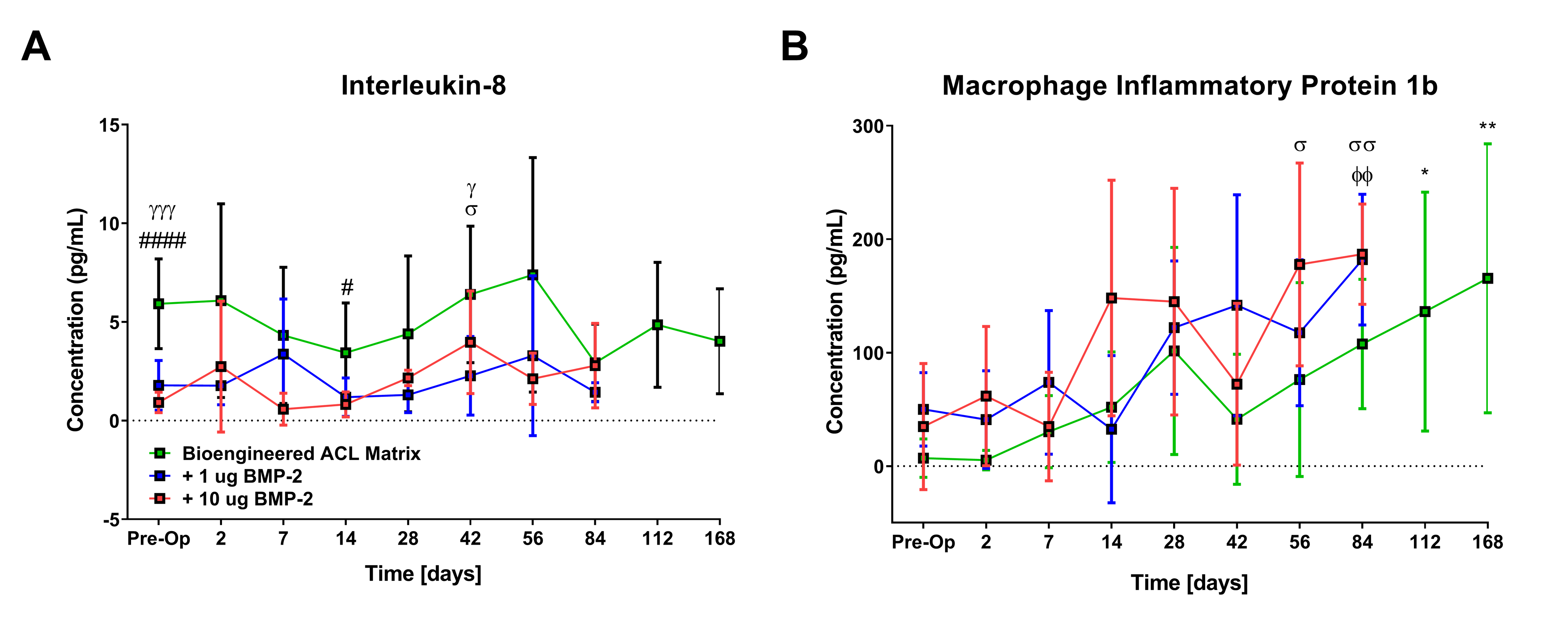

Supplement: S3 Fig — Evaluation of systemic IL-8 A) and MIP-1b B) levels before and after ACL reconstruction. (σ = significant difference for 10 μg group in comparison to pre-operative levels; γ = significant difference between control and 1 μg BMP-2; # = significant difference between control and 10 μg BMP-2; Φ = 1 ug group analysis between pre-operative levels; * = control pre-operative vs time point cytokine values). (TIF) [file pone.0227181.s003.tif]
