# Supplementary material for: Evaluation of a bioengineered ACL matrix’s osteointegration with BMP-2 supplementation
Source: PLoS One. 2020 Jan 7;15(1):e0227181. doi: 10.1371/journal.pone.0227181 (PMC6946545; doi:10.1371/journal.pone.0227181)
Supplement: S5 File — (DOCX) [file pone.0227181.s015.docx]

# Supporting Information

**Cytokine Analysis**

For the bioengineered ACL matrix, IL-8 levels dropped off from pre-operative levels and peaked at Day 56 (S2 Fig). Time of blood sampling did not significantly influence IL-8 levels for the bioengineered ACL matrix. IL-8 levels peaked at day 7 and day 56 in the +1 µg BMP-2 treatment, but the time of blood sampling did not significantly influence these levels. In the +10 µg BMP-2 treatment, IL-8 levels peaked at days 2 and 42. At day 42, IL-8 levels were significantly greater than found pre-operatively (p-value = 0.032). In comparison to the bioengineered ACL matrix alone, IL-8 levels were significantly lower at day 42 for grafts supplemented with 1 µg of BMP-2 (p-value = 0.0368), and significantly lower at day 14 for grafts supplemented with 10 µg of BMP-2 (p-value = 0.0239). However, the results must be taken with caution due to significant differences in the pre-operative levels between all groups.

MIP-1b cytokine levels fluctuated in all groups with peaks seen at days 14, 28, and 56 (S2 Fig). For the bioengineered ACL matrix, MIP-1b levels were significantly elevated at Day 112 and 168. MIP-1b levels were significantly elevated at day 84 in the +1 µg BMP-2 group. MIP-1b levels were significantly elevated at day 56 and 84 in the +10 µg BMP-2 group. In comparing MIP-1b levels of the bioengineered ACL matrix versus +1 µg or +10 µg BMP-2, no significant difference at individual timepoints was found.

Evaluation of systemic cytokine levels demonstrated the potential use of MIP-1b as a biomarker to detect inflammation in the knee joint. The upregulation of MIP-1b from day 42 to day 168 for the bioengineered ACL matrix may be due to progressive inflammation after graft rupture. Cooper et al.’s previous ACL reconstruction evaluation demonstrated that the grafts were all intact at day 28 but 67% of grafts ruptured at day 84 [1]. Therefore, it seems that the rupture of the grafts may occur between days 42 and 84 based on the cytokine data. An understanding of the role of BMP-2 supplementation on cytokine levels is difficult to distinguish and likely due to measuring the systemic inflammatory state rather than the inflammatory state of the knee joint. Future analysis of cytokines for ACL reconstruction should evaluate the synovial fluid to gain a better understanding of the local inflammatory response during healing.

# References

1. Cooper JA, Sahota JS, Gorum WJ, Carter J, Doty SB, Laurencin CT. Biomimetic tissue-engineered anterior cruciate ligament replacement. Proc Natl Acad Sci U S A. 2007;104: 3049–54. doi:10.1073/pnas.0608837104
